# Quinoline-3-carboxamide Derivatives as Potential Cholesteryl Ester Transfer Protein Inhibitors

**DOI:** 10.3390/molecules17055497

**Published:** 2012-05-09

**Authors:** Wen-Yan Li, Xu-Qiong Xiong, Dong-Mei Zhao, Yu-Fang Shi, Zhi-Heng Yang, Chao Yu, Pei-Wei Fan, Mao-Sheng Cheng, Jing-Kang Shen

**Affiliations:** 1 Key Laboratory of Structure-Based Drugs Design & Discovery of Ministry of Education, School of Pharmaceutical Engineering, Shenyang Pharmaceutical University, Shenyang 110016, China; 2 Central Research Institute, Shanghai Pharmaceuticals Holding Co., Ltd., Shanghai 201203, China

**Keywords:** CETP inhibitors, CETP, quinoline-3-carboxamide derivatives, synthesis, high density lipoprotein cholesterol

## Abstract

A series of novel quinoline-3-carboxamide derivatives **10**–**17** and **23**–**27** were designed and synthesized as cholesteryl ester transfer protein (CETP) inhibitors. All of them exhibited activity against CETP. Particularly, compounds **24** and **26** displayed the best activity against CETP with the same inhibitory rate of 80.1%.

## 1. Introduction

**Figure 1:**
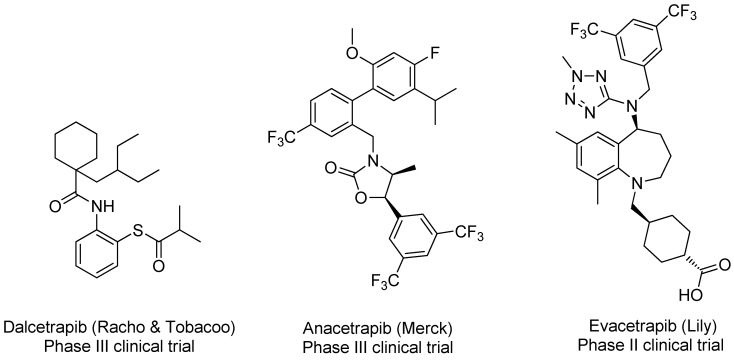
Structures of current CETP inhibitors.

Coronary heart disease (CHD), a leading cause of death around the World [1], has an inverse relationship with serum high density lipoprotein (HDL) cholesterol levels [2]. Raising of HDL cholesterol levels provides a potential therapeutic benefit for CHD patients [3,4]. Cholesteryl ester transfer protein (CETP) is a plasma glycoprotein which plays an important role in decreasing HDL-C level and increasing LDL-C level [5]. Inhibition of CETP may be a new therapy for atherosclerosis [6,7,8,9,10]. The research on CETP inhibitors such as dalcetrapib, anacetrapib and evacetrapib (Figure 1) has become a new hotspot for the treatment of CHD [11,12,13].

**Figure 2:**
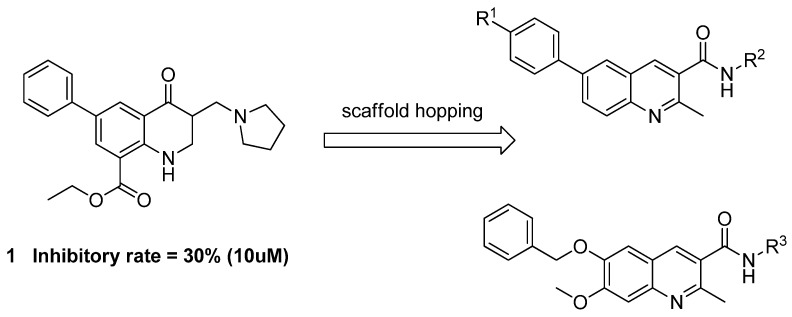
Scaffold hopping from tetrahydroquinolone into quinoline analogues.

In our lab a series of 2,3-dihydro-4-tetrahydroquinolones were found to have potent CETP inhibitory activity by virtual screening, including lead-like rule, clustering analysis, biological activity spectra prediction, ADME/Tox prediction and synthetic feasibility prediction [14]. Compound **1**, a best effective CETP inhibitor among these derivatives, showed 30% inhibitory rate against CETP at 10 μ M in a buffer assay. In this study, our primary objective was to optimize the potency of compound **1** against CETP and obtain more potent CETP inhibitors. Based on the scaffold hopping strategy, 2,3-dihydro-4-tetrahydroquinolone was changed to a quinoline ring while retaining the aryl group at the 6 position (Figure 2). 6-Arylquinoline-3-carboxamide derivatives **10**–**17** were thus designed and synthesized. The crystal structure of CETP reveals a long continuous tunnel (60 Å) with a very large volume (2,560 Å^3^) [15], so in order to make the structures bind nicely with CETP in the bulky active cavity, the phenyl ring substituent was replaced by larger and more flexible 6-benzyloxy and 7-methoxy groups to give the derivatives **23**–**27** which were also synthesized. All of target compounds **10**–**17**, **23**–**27** were evaluated their CETP inhibitory activity by a radioisotope-based assay. 

## 2. Result and Discussion

### 2.1. Chemistry

**Scheme 1:**
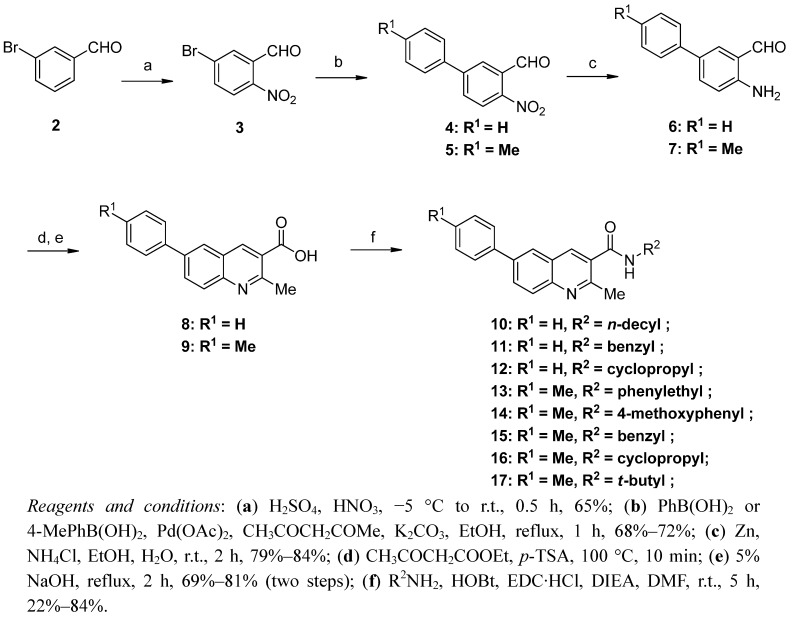
The synthesis of 6-phenylquinoline-3-carboxamide derivatives **10**–**17**.

The synthetic pathway to the 6-phenylquinoline-3-carboxamide derivatives is shown in Scheme 1. Compound **3** was obtained in 65% yield by treatment of 3-bromobenzaldehyde **2** with violet acid (H_2_SO_4_:HNO_3_ = 10:1, V/V). Suzuki coupling of aryl bromide **3** with phenylboronic acid or 4-methylphenylboronic acid gave compounds **4** or **5**, which were reduced with zinc dust to provide substituted *o*-amino benzaldehydes **6** or **7**. 3-Quinolinecarboxylic acids **8** or **9** was prepared by cyclization of **6** or **7** with ethyl acetoacetate and *p*-tolylsulfonic acid at 100 °C and then hydrolyzed with 5% NaOH solution. Coupling of **8** or **9** with commercially available amines afforded the target compounds **10**–**17**.

**Scheme 2:**
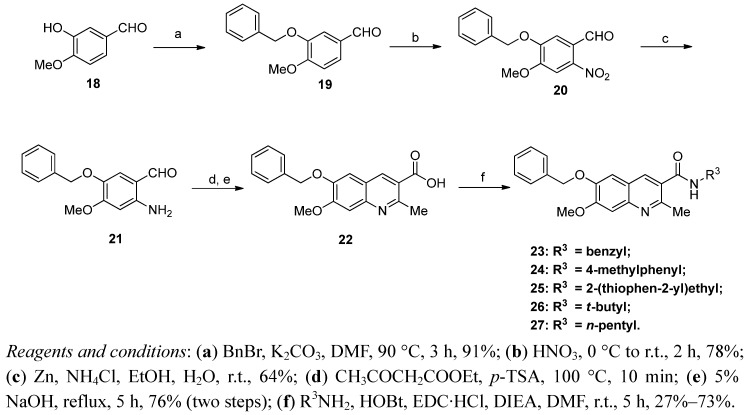
The synthesis of 6-benzyloxy-7-methoxyquinoline-3-carboxamide derivatives **23**–**27**.

The preparation of the 6-benzyloxy-7-methoxyquinoline-3-carboxamide derivatives is shown in Scheme 2. Alkylation of commercially available isovanillin (**18**) with benzyl bromide and potassium carbonate at 90 °C in DMF produced compound **19**, which was nitrated with nitric acid at 0 °C to afford **20**. The later steps were same as the synthesis of the 6-phenylquinoline-3-carboxamide derivatives.

### 2.2. Biological Activity

*In vitro* inhibitory activity of all target compounds **10**–**17**, **23**–**27** against CETP was evaluated by a radioisotope-based assay. The inhibition (%) results are presented in Table 1. All the newly synthesized derivatives exhibited considerable CETP inhibitory activity (inhibitory rates: 20.7%–80.1%). Particularly, *p*-tolyl amide **24** and *t*-butyl substituted amide **26** exhibited the best inhibitory activity towards CETP, with the same inhibitory rate of 80.1%, which is approximately the same activity of the positive control dalcetrapib. Substitution at 6 and 7 positions of the quinoline-3-carboxamide scaffold was considered a determining factor in the activity. Indeed, compounds **26**, **24** and **23** with 6-benzyloxy-7-methoxy groups showed better activity than the corresponding substituted 6-phenyl analogues **17**, **14** and **11**, respectively. We speculate that the bulky 6-benzyloxy-7-methoxy group packs nicely in the large hydrophobic cavity of CETP. In addition, compounds **14** and **24** with a substituted aryl group on the 3-carboxamide nitrogen atom showed higher potency than other corresponding alkyl analogues in the two chemical classes.

## 3. Experimental

### 3.1. General

All melting points were obtained on a Büchi Melting Point B-540 apparatus (Büchi Labortechnik, Flawil, Switzerland) and were uncorrected. Mass spectra (MS) were taken in ESI mode on Agilent 1100 LC-MS (Agilent, Palo Alto, CA, USA). Nuclear magnetic resonance spectroscopy was performed using Bruker ARX-300, 300 MHz spectrometers (Bruker Bioscience, Billerica, MA, USA) with TMS as an internal standard. IR spectra (KBr disks) were recorded with a Bruker IFS-55 instrument (Bruker). All the materials were obtained from commercially available sources and used without further purification, unless otherwise specified. Yields were not optimized. Compound **21** was synthesized according to the literature [16,17].

*5-Bromo-2-nitrobenzaldehyde* (**3**). Compound **2** (27.1 g, 0.15 mol) was slowly added dropwise to violet acid (150 mL, H_2_SO_4_:HNO_3_ = 10:1, V/V) cooled to −5 °C and then stirred at room temperature for 0.5 h. The reaction mixture was poured into ice water to give a white precipitate which was filtered off and purified by recrystallization from 5:1 petroleum ether/ethyl acetate to give the desired product **3** (21.7 g, 65%) as a off-white solid; m.p. 60.5–61.9 °C. ^1^H-NMR (CDCl_3_) δ: 7.89 (1H, dd, *J_1_* = 8.7 Hz, *J_2_* = 2.1 Hz), 8.03 (1H, d, *J* = 8.7 Hz), 8.06 (1H, d, *J* = 2.1 H), 10.41 (1H, s).

*4-Nitrobiphenyl-3-benzaldehyde* (**4**). To a solution of **3** (22.7 g, 0.1 mol) in alcohol (150 mL) and K_2_CO_3_ (1 M) (150 mL) was added phenylboronic acid (16.8 g, 0.14 mol) and then Pd(AcO)_2_ (0.10 g, 0.4 mmol) and acetylacetone (0.3 mL, 1.2 mmol). The mixture was refluxed for 1 h and cooled to room temperature. The solution was concentrated and quenched with water, then extracted with ethyl acetate. The organic layer was washed with water and brine, and then dried over Na_2_SO_4_. Solvent was removed under reduced pressure and the resulting residue was purified by column chromatography (25% EtOAc/petroleum ether, silica) to provide the title compound (16.3 g, 72% yield) as a yellow solid; m.p. 71.0–73.4 °C. ^1^H-NMR (CDCl_3_) δ: 7.49 (2H, t, *J* = 6.3 Hz), 7.50 (1H, t, *J* = 6.3 Hz), 7.66 (2H, d, *J* = 8.1 Hz), 7.93 (1H, d, *J* = 8.4 Hz), 8.1 (1H, d, *J* = 2.1 Hz), 8.22 (1H, d, *J* = 8.4 Hz), 10.51 (1H, s). 

*4′-Methyl-4-nitrobiphenyl-3-benzaldehyde* (**5**). **5** was obtained as a yellow solid (67.7% yield) from compound **3** as described for **4**; m.p. 54.2–58.4 °C. ^1^H-NMR (CDCl_3_) δ: 2.43 (3H, s), 7.32 (2H, d, *J* = 8.1 Hz), 7.56 (2H, d, *J* = 8.1 Hz), 7.92 (1H, d, *J* = 8.4 Hz), 8.1 (1H, d, *J* = 2.1 Hz), 8.20 (1H, d, *J* = 8.4 Hz), 10.52 (1H, s).

*4-Aminobiphenyl-3-benzaldehyde* (**6**). To a solution of **4** (5.0 g, 22 mmol) in water (100 mL) and alcohol (33 mL) was added ammonium chloride (7.1 g, 132 mmol) and then zinc dust (17.2 g, 264 mmol). The mixture was stirred at room temperature for 2 h and filtered. The filtrate was concentrated and quenched with water, then extracted with ethyl acetate. The organic layer was washed with water and brine and then dried over Na_2_SO_4_. Solvent was removed under reduced pressure and obtained **6** (3.4 g, 79% yield) as a yellow solid used directly to the next step without any purification; m.p. 124.5–125.8 °C. ^1^H-NMR (CDCl_3_) δ: 6.19 (2H, s), 6.77 (1H, d, *J* = 8.5 Hz), 7.33 (2H, t, *J* = 7.3 Hz), 7.45 (2H, t, *J* = 7.3 Hz), 7.56 (1H, d, *J* = 7.1 Hz), 7.61 (1H, dd, *J_1_* = 2.2 Hz, *J_2_* = 8.5 Hz), 7.74 (1H, d, *J* = 2.2 Hz), 9.98 (1H,s). MS *m/z*: 198.1 [M+H]^+^.

*4′-Methyl-4-aminobiphenyl-3-benzaldehyde* (**7**). Compound **7** was obtained from compound **5** as a yellow solid (84.8% yield) as described for **6** and used directly to the next step without any purification; m.p. 148.2–149.4 °C. MS *m/z*: 212.1 [M+H]^+^.

*6-Phenyl-2-methylquinoline-3-carboxylic acid* (**8**). Compound **6** (3.4 g, 17 mmol) was mixed with acetoacetic ester (2.3 g, 17 mmol) and *p*-tolylsulfonic acid (2.9 g, 17 mmol). The mixture was heated to 100 °C for 10 min and cooled to room temperature. Next 10% NaOH (20 mL) was added and stirred for 30 min to give a yellow precipitate. The precipitate was isolated by filtration and washed with water to give **8** as a yellow solid. Without any purification, the solid was dissolved in 5% NaOH (100 mL). The solution was refluxed for 2 h and cooled to room temperature. The reaction mixture’s pH value was adjusted to 2 with 6 N hydrochloric acid giving a greyish white precipitate. The precipitate was filtered and washed with water to pH 7. The filter cake was dried to give **8** (3.1 g, 69% yield for two steps) as a greyish white solid; m.p. 187.8–188.9 °C. ^1^H-NMR (DMSO-*d*_6_) δ: 2.96 (3H, s), 7.46 (1H, t, *J* = 7.1 Hz), 7.55 (2H, t, *J* = 7.2 Hz), 7.85 (2H, d, *J* = 7.4 Hz), 8.30–8.19 (2H, m), 8.53 (1H, d, *J* = 8.8 Hz), 9.08 (1H, s), 11.20 (1H, s). MS *m/z*: 262.1 [M−H]^−^.

*2-Methyl-6-(p-tolyl)quinoline-3-carboxylic acid* (**9**). Compound **9** was obtained from compound **7** as a greyish white solid (80.9% yield) as described for **8**; m.p. 201.8–203.4 °C. ^1^H-NMR (CDCl_3_) δ: 2.45 (3H, s), 2.96 (3H, s), 7.30 (2H, d, *J* = 8.0 Hz), 7.46 (2H, d, *J* = 7.9 Hz), 8.28–8.17 (2H, m), 8.49 (1H, d, *J* = 8.8 Hz), 9.01 (1H, s), 11.1 (1H, s).

*3′-O-Benzylisovanillin* (**19**). Compound **19** was obtained as a white solid (92% yield) according to the literature [17]; m.p. 50.1–52.9 °C. ^1^H-NMR (CDCl_3_) δ: 3.96 (3H, s), 5.22 (2H, s), 7.01 (1H, d, *J* = 8.1 Hz), 7.25–7.49 (7H, m), 9.80 (1H, s).

*5-Benzyloxy-4-methoxy-2-nitrobenzaldehyde* (**20**). Compound **20** was obtained as a white solid (78% yield) according to the literature [16]; m.p. 131.8–133.0 °C. ^1^H-NMR (CDCl_3_) δ: 4.01 (3H, s), 5.25 (2H, s), 7.44 (6H, m), 7.63 (1H, s), 10.42 (1H, s).

*5-Benzyloxy-4-methoxy-2-aminobenzaldehyde* (**21**). Compound **21** was obtained from compound **20** as a yellow solid (64% yield) as described for **6** and used directly to the next step without any purification; m.p. 180–184 °C. MS *m/z*: 258.1 [M+H]^+^.

*6-Benzyloxy-7-methoxy-2-methylquinoline-3-carboxylic acid* (**22**). Compound **22** was obtained from compound **21** as a greyish white solid (76% yield) as described for **8**; m.p. 274.2–278.4 °C. ^1^H-NMR (DMSO-*d*_6_) 2.82 (3H, s), 4.02 (3H, s), 5.27 (2H, s), 7.37–7.70 (7 H, m), 8.66 (1H, s).

### 3.2. General Procedure for the Synthesis of Quinoline-3-carboxamide Derivatives ***10**–**17**, **23**–**27***

To a solution of **8**, **9**, **22** (1 mmol) in dry DMF (10 mL) was added 1-hydroxybenzotrizole (HOBt) (1.5 mmol) and 1-ethyl-3-(3-dimethylaminopropyl)carbodiimide hydrochloride (EDC·HCl, 1.5 mmol). The mixture was stirred at room temperature for 2 h, and then the corresponding amine (2.0 mmol) and DIEA (2.0 eq) were added. The reaction mixture was stirred at room temperature for 5 h, poured into ice water. The precipitate was filtered, washed with water, and then recrystallized with ethyl acetate or purified by column chromatography (silica gel) to give **10**–**17**, **23**–**27**.

*N-Decyl-2-methyl-6-phenylquinoline-3-carboxamide* (**10**). White solid, 84% yield, m.p. 109.1–109.5 °C. ^1^H-NMR (DMSO-*d*_6_) δ: 0.85 (3H, t), 1.25–1.32 (14H, m), 1.55 (2H, t), 2.68 (3H, s), 3.28 (2H, t), 7.42 (1H, t, *J* = 7.3 Hz), 7.53 (2H, t, *J* = 7.2 Hz), 7.81 (2H, d, *J* = 7.5 Hz), 8.02 (1H, d, *J* = 8.8 Hz), 8.10 (1H, dd, *J_1_* = 8.8 Hz, *J_2_* = 2.0 Hz), 8.29 (1H, d, *J* = 1.8 Hz), 8.33 (1H, s), 8.58 (1H, s). ^13^C-NMR (DMSO-*d*_6_) δ: 13.9, 22.1, 23.3, 26.4, 28.7, 29.0, 31.3, 125.3, 125.8, 127.0, 128.6, 129.1, 129.3, 131.4, 134.6, 137.7, 139.2, 146.3, 155.8, 167.7. HR-MS *m/z*: 403.2838 (calcd for C_27_H_35_N_2_O [M+H]^+^: 403.2744). IR (KBr) cm^−1^: 3230, 3055, 2917, 2851, 1634, 1600, 1578, 837, 755, 699.

*N-Benzyl-2-methyl-6-phenylquinoline-3-carboxamide* (**11**). White solid, 52% yield, m.p. 168.9–171.1 °C. ^1^H-NMR(DMSO-*d_6_*): 2.70 (3H, s), 4.53 (2H, d, *J* = 5.8 Hz), 7.26–7.30 (1H, m), 7.35–7.45 (5H, m), 7.54 (2H, t, *J* = 7.3 Hz), 7.82 (1H, d, *J* = 7.4 Hz), 8.03 (1H, d, *J* = 8.7 Hz), 8.11 (1H, dd, *J_1_* = 8.7 Hz, *J_2_* = 1.9 Hz), 8.33 (1H, d, *J* = 1.7 Hz), 8.43 (1H, s), 9.17 (1H, t, *J* = 5.9 Hz). ^13^C-NMR (DMSO-*d*_6_) δ: 23.4, 42.6, 125.4, 125.8, 126.9, 127.0, 127.3, 127.9, 128.4, 128.6, 129.1, 129.4, 130.9, 134.8, 137.8, 139.1, 139.2, 146.4, 155.9, 167.8. HR-MS *m/z*: 353.1658 (calcd for C_24_H_21_N_2_O [M+H]^+^: 353.1648). IR (KBr) cm^−1^: 3241, 3066, 1629, 1598, 1573, 1023, 838, 755, 699. 

*N-Cyclopropyl-2-methyl-6-phenylquinoline-3-carboxamide* (**12**). White solid, 22% yield, m.p. 186.7–187.1 °C. ^1^H-NMR(DMSO-*d_6_*) δ: 0.51–0.64 (2H, m), 0.69–0.75 (2H, m), 2.68 (3H, s), 2.87–2.93 (1H, m), 7.42 (1H, t, *J* = 7.4 Hz), 7.53 (2H, t, *J* = 9.1 Hz), 7.81 (2H, d, *J* = 7.4 Hz), 8.01 (1H, d, *J* = 8.7 Hz), 8.10 (1H, dd, *J_1_* = 8.8 Hz, *J_2_* = 1.9 Hz), 8.28 (1H, d, *J* = 1.7 Hz), 8.33 (1H, s), 8.65 (1H, s). ^13^C-NMR (DMSO-*d*_6_) δ: 5.7, 22.8, 23.3, 125.3, 125.7, 127.0, 127.8, 128.6, 129.1, 129.4, 131.0, 134.7, 137.8, 139.2, 146.3, 155.9, 168.9. HR-MS *m/z*: 303.1564 (calcd for C_20_H_19_N_2_O [M+H]^+^: 303.1492). IR (KBr) cm^−1^: 3290, 3034, 1643, 1594, 1579, 1529, 696.

*N-(2-Phenylethyl)-2-methyl-6-(p-tolyl)quinoline-3-carboxamide* (**13**). Yellow solid, 64% yield, m.p. 174.5–175.6 °C. ^1^H-NMR (DMSO-*d_6_*) δ: 2.37 (3H, s), 2.59 (3H, s), 2.89 (2H, t, *J* = 7.1 Hz), 3.55 (2H, t, *J* = 6.5 Hz), 7.22–7.35 (7H, m), 7.72 (2H, d, *J* = 7.8 Hz), 7.99 (1H, d, *J* = 8.7 Hz), 8.07 (1H, d, *J* = 8.7 Hz), 8.22 (1H, s), 8.26 (1H, s), 8.68 (1H, s). ^13^C-NMR (DMSO-*d*_6_) δ: 20.7, 23.2, 34.9, 40.5, 124.8, 125.8, 126.1, 126.8, 128.3, 128.5, 128.7, 129.2, 129.7, 131.2, 134.5, 136.3, 137.3, 137.7, 139.3, 146.2, 155.7, 167.4. HR-MS *m/z*: 381.1971 (calcd for C_26_H_25_N_2_O [M+H]^+^: 381.1961). IR (KBr) cm^−1^: 3237, 3063, 3028, 2936, 1631, 1599, 1574, 1491, 811, 748, 700.

*N-(4-methoxyphenyl)-2-methyl-6-(p-tolyl) quinoline-3-carboxamide* (**14**). Yellow solid, 84 % yield, m.p. 221.0–221.8 °C. ^1^H-NMR (DMSO-*d_6_*) δ: 2.37 (3H, s), 2.74 (3H, s), 3.76 (3H, s), 6.96 (2H, d, *J* = 8.9 Hz), 7.34 (2H, d, *J* = 8.0 Hz), 7.67–7.74 (4H, m), 8.04 (1H, d, *J* = 8.7 Hz), 8.12 (1H, dd, *J_1_* = 8.8 Hz, *J_2_* = 1.7 Hz), 8.31 (1H, d, *J* = 1.3 Hz), 8.51 (1H, s), 10.49 (1H, s). ^13^C-NMR (DMSO-*d*_6_) δ: 20.7, 23.4, 55.2, 113.9, 121.2, 124.9, 125.8, 126.8, 128.5, 129.5, 129.7, 131.1, 132.2, 135.1, 136.2, 137.3, 137.8, 146.3, 155.6, 166.0. HR-MS *m/z*: 383.1842 (calcd for C_25_H_23_N_2_O_2_ [M+H]^+^: 383.1754). IR (KBr) cm^−1^: 3247, 3029, 1648, 1595, 1525,1512, 1245, 813. 

*N-Benzyl-2-methyl-6-(p-tolyl)quinoline-3-carboxamide* (**15**). Yellow solid, 31 % yield, m.p. 181.8–182.4 °C. ^1^H-NMR(DMSO-*d_6_*) δ: 2.31 (3H, s), 2.69 (3H, s), 4.53 (2H, d), 7.46–7.24 (7H, m), 7.73 (2H, d, *J* = 7.9 Hz), 8.01 (1H, d, *J* = 8.7 Hz), 8.08 (1H, d, *J* = 9.1 Hz), 8.29 (1H, s), 8.41 (1H, s), 9.15 (1H, m). ^13^C-NMR (DMSO-*d*_6_) δ: 20.7, 23.4, 42.6, 124.9, 125.8, 126.8, 126.9, 127.3, 128.4, 128.5, 129.3, 129.7, 130.9, 134.8, 136.3, 137.3, 137.7, 139.2, 146.3, 155.7, 167.9. HR-MS *m/z*: 367.1892 (calcd for C_25_H_23_N_2_O [M+H]^+^: 367.1805). IR (KBr) cm^−1^: 3273, 2919, 1631, 1594, 1540, 808.

*N-Cyclopropyl-2-methyl-6-(p-tolyl) quinoline-3-carboxamide* (**16**). White solid, 43% yield, m.p. 220.0–220.9 °C. ^1^H-NMR (DMSO-*d_6_*) δ: 0.58 (2H, s), 0.73 (2H, s), 2.37 (3H, s), 2.67 (3H, s), 2.88 (1H, s), 7.34 (2H, d, *J* = 5.6 Hz), 7.71 (2H, d, *J* = 5.4 Hz), 8.01 (1H, s), 8.06 (1H, s), 8.24 (1H, s) 8.30 (1H, s), 8.62 (1H, s). ^13^C-NMR (DMSO-*d*_6_) δ: 20.7, 23.3, 23.5, 32.2, 40.0, 50.8, 124.8, 125.9, 126.8, 128.5, 129.1, 129.7, 131.4, 134.5, 136.3, 137.2, 137.6, 146.2, 155.7, 167.3. HR-MS *m/z*: 317.1633 (calcd for C_21_H_21_N_2_O [M+H]^+^: 317.1648). IR (KBr) cm^−1^: 3279, 2962, 2869, 1632, 1542, 806.

*N-(tert-Butyl)-2-methyl-6-(p-tolyl)quinoline-3-carboxamide* (**17**). White solid, 24% yield, m.p. 181.7–183.6 °C. ^1^H-NMR (CDCl_3_) δ: 1.47 (9H, s), 2.36 (3H, s), 2.78 (3H, s), 7.23 (2H, d, *J* = 8.0 Hz), 7.51 (2H, d, *J* = 8.1 Hz), 7.89 (2H, d, *J* = 9.4 Hz), 8.01 (1H, d, *J* = 9.3 Hz), 8.08 (1H, s). ^13^C-NMR (DMSO-*d*_6_) δ: 20.7, 23.1, 28.5, 50.9, 124.8, 125.9, 126.7, 128.5, 128.9, 129.7, 132.3, 134.1, 136.3, 137.2, 137.5, 146.1, 155.5, 167.7. HR-MS *m/z*: 333.2041 (calcd for C_22_H_25_N_2_O [M+H]^+^: 333.1961). IR (KBr) cm^−1^: 3252, 2967, 1647, 1600, 1548, 929, 813, 802.

*6-(Benzyloxy)-7-methoxy-2-methyl-N-(benzyl)quinoline-3-carboxamide* (**23**). White solid, 33% yield, m.p. 214.6–215.3 °C. ^1^H-NMR (DMSO-*d_6_*) δ: 2.60 (3H, s), 3.91 (3H, s), 4.46 (2H, d, *J* = 5.7 Hz), 5.19 (2H, s), 7.25 (1H, s), 7.33–7.41 (8H, m), 7.46–7.49 (3H, m), 8.12 (1H, s), 9.00 (1H, d, *J* = 7.1 Hz). ^13^C-NMR (DMSO-*d*_6_) δ: 23.2, 42.5, 55.7, 69.9, 107.1, 107.2, 120.6, 126.8, 127.2, 128.0, 128.3, 128.5, 133.1, 136.5, 139.4, 144.3, 148.2, 153.0, 153.2, 168.2. HR-MS *m/z*: 413.1870 (calcd for C_26_H_25_N_2_O_3_ [M+H]^+^: 413.1860). IR (KBr) cm^−1^: 3283, 3029, 2964, 2935, 1634, 1540, 748, 703.

*6-(Benzyloxy)-7-methoxy-2-methyl-N-(4-methylphenyl)quinoline-3-carboxamide* (**24**). White solid, 44% yield, m.p. 202.1–202.8 °C. ^1^H-NMR (DMSO-*d_6_*) δ: 2.28 (3H, s), 2.93 (3H, s), 4.02 (3H, s), 5.28 (2H, s), 7.16–7.81 (11H, m), 8.93 (1H, s), 10.78 (1H, s). ^13^C-NMR (DMSO-*d*_6_) δ: 23.4, 55.2, 113.9, 121.2, 125.4, 125.7, 127.0, 127.9, 128.7, 129.1, 129.6, 131.1, 132.2, 135.1, 137.9, 139.2, 146.4, 155.6, 155.8, 166.0. HR-MS *m/z*: 413.1869 (calcd for C_26_H_25_N_2_O_3_ [M+H]^+^: 413.1860). IR (KBr) cm^−1^: 3261, 3033, 2927, 1648, 1522, 752.

*6-Benzyloxy-7-methoxy-2-methyl-N-(2-(thiophen-2-yl)ethyl)quinoline-3-carboxamide* (**25**). White solid, 27% yield, m.p. 178.8–179.5 °C. ^1^H-NMR (DMSO-*d*_6_) δ: 2.58 (3H, s), 3.09 (2H, t), 3.48–3.57 (2H, q), 3.93 (3H, s), 5.22 (2H, s), 6.93–7.01 (2H, m), 7.33–7.46 (6H, m), 7.48–7.55 (2H, m), 8.05 (1H, s), 8.63 (1H, s). ^13^C-NMR (DMSO-*d*_6_) δ: 23.1, 29.2, 40.7, 55.7, 69.9, 107.1, 107.2, 120.5, 124.1, 125.3, 126.9, 128.0, 128.1, 128.4, 128.5, 132.9, 136.5, 141.5, 144.2, 148.2, 152.9, 153.2, 168.2. HR-MS *m/z*: 433.1681 (calcd for C_25_H_25_O_3_N_2_S [M+H]^+^: 433.1580). IR (KBr) cm^−1^: 3311, 3081, 2966, 2924, 2863, 1636, 1536. 

*6-Benzyloxy-7-methoxy-2-methyl-N-(tert-butyl)quinoline-3-carboxamide* (**26**). White solid, 35% yield, m.p. 136.8–138.1 °C. ^1^H-NMR (DMSO-*d*_6_) δ: 1.39 (9H, s). 2.59 (3H, s), 3.93 (3H, s), 5.20 (2H, s), 7.33–7.52 (7H, m), 7.99 (1H, s), 8.06 (1H, s). ^13^C-NMR (DMSO-*d*_6_) δ: 22.8, 28.5, 50.8, 55.7, 69.7, 107.1, 120.6, 128.0, 128.5, 132.6, 136.5, 143.9, 148.0, 152.7, 152.9, 168.1. HR-MS *m/z*: 379.2025 (calcd for C_23_H_27_N_2_O_3_ [M+H]^+^: 379.2016). IR (KBr) cm^−1^: 3227, 3029, 2965, 1666, 1604, 1503, 748.

*6-Benzyloxy-7-methoxy-2-methyl-N-(n-pentyl)quinoline-3-carboxamide* (**27**). Yellow solid, 73% yield, m.p. 168.0–168.5 °C. ^1^H-NMR (DMSO-*d*_6_) δ: 0.89 (3H, t, *J* = 6.6 Hz), 1.33–1.34 (4H, m), 1.51–1.56 (2H, m), 2.61 (3H, s), 3.24–3.33 (2H, m), 3.93 (3H, s), 5.21 (2H, s), 7.35–7.53 (7H, s), 8.05 (1H, s), 8.45 (1H, t, *J* = 5.3 Hz). ^13^C-NMR (DMSO-*d*_6_) δ: 13.9, 21.8, 23.0, 28.6, 28.7, 55.7, 69.9, 107.1, 107.2, 120.6, 128.0, 128.5, 128.8, 132.8, 136.5, 144.1, 148.1, 152.9, 153.1, 168.1. HR-MS *m/z*: 393.2183 (calcd for C_24_H_29_N_2_O_3_ [M+H]^+^: 393.2173). IR (KBr) cm^−1^: 3283, 2929, 2857, 1634, 1500.

### 3.3. CETP Inhibition Assay

CETP activity was determined by detecting the exchange of radioactive cholesteryl ester between labelled HDL and unlabelled LDL [18]. Briefly, fresh rabbit serum containing CETP was incubated in TSE buffer (50 mM Tris, 50 mM NaCl, 2 mM EDTA and 1% bovine serum albumin) containing 10 μmol/L test compounds dissolved in dimethyl sulfoxide [final concentration of both rabbit serum and dimethyl sulfoxide in incubation mixture was 0.5% in 300 μL volume] for 4 h at 37 °C. Then incubated for another 16 h at 37 °C with 0.5 μL [^3^H]cholesteryl ester-labeled HDL and 5 μL unlabelled LDL in 600 μL volume. LDL was precipitated with dextran sulfate (final concentration: 0.027%) and MgCl_2_ (final concentration: 27 mM) for 30 min. Centrifuged at 5,000 g and 4 °C for 30 min to get the supernatant, and its radioactivity was measured in a liquid scintillation counter (Wallac 1410, Pharmacia, Uppsala, Sweden). The CETP activity was determined by the decrease in radioactivity *versus* that of a blank without serum.

## 4. Conclusions

In conclusion, a series of novel quinoline-3-carboxamide derivatives **10**–**17** and **23**–**27** were synthesized and their CETP inhibitory activity evaluated. Generally, compounds substituted with 6-benzyloxy-7-methoxy groups possessed more potent CETP inhibitory activity. Compounds **24** and **26** exhibited promising inhibitory activity of CETP (80.1%) compared with the lead compound **1** (30%). As a novel CETP inhibitor scaffold, further structural modifications of the quinoline-3-carboxamide moiety are progressing in our lab.

## Figures and Tables

**Table 1:** CETP inhibitory rate of the target compounds (**10**–**17**, **23**–**27**).

Compd.	Inhibition (%) ^a^	Compd.	Inhibition (%) ^a^
**10**	30.0 ± 1.2	**17**	35.2 ± 0.7
**11**	34.2 ± 0.7	**23**	51.5 ± 2.6
**12**	41.8 ± 1.5	**24**	80.1 ± 1.3
**13**	31.5 ± 1.1	**25**	29.8 ± 2.5
**14**	71.7 ± 2.2	**26**	80.1 ± 0.8
**15**	52.5 ± 2.1	**27**	20.7 ± 2.3
**16**	36.7 ± 1.9	Dalcetrapib ^b^	82.5 ± 2.0

^a^ Values represent the percent inhibition of CETP at 10 µM of the test compounds and are the average of three independent experiments; ^b^ The positive control.
